# The Complete Chloroplast and Mitochondrial Genome Sequences of *Boea hygrometrica*: Insights into the Evolution of Plant Organellar Genomes

**DOI:** 10.1371/journal.pone.0030531

**Published:** 2012-01-23

**Authors:** Tongwu Zhang, Yongjun Fang, Xumin Wang, Xin Deng, Xiaowei Zhang, Songnian Hu, Jun Yu

**Affiliations:** 1 James D. Watson Institute of Genome Sciences, Zhejiang University, Hangzhou, China; 2 CAS Key Laboratory of Genome Sciences and Information, Beijing Institute of Genomics, Chinese Academy of Sciences, Beijing, China; 3 CAS Key Laboratory of Photosynthesis and Molecular Physiology, Research Center of Plant Molecular and Development Biology, Institute of Botany, Chinese Academy of Sciences, Beijing, China; J. Craig Venter Institute, United States of America

## Abstract

The complete nucleotide sequences of the chloroplast (cp) and mitochondrial (mt) genomes of resurrection plant *Boea hygrometrica* (*Bh*, Gesneriaceae) have been determined with the lengths of 153,493 bp and 510,519 bp, respectively. The smaller chloroplast genome contains more genes (147) with a 72% coding sequence, and the larger mitochondrial genome have less genes (65) with a coding faction of 12%. Similar to other seed plants, the *Bh* cp genome has a typical quadripartite organization with a conserved gene in each region. The *Bh* mt genome has three recombinant sequence repeats of 222 bp, 843 bp, and 1474 bp in length, which divide the genome into a single master circle (MC) and four isomeric molecules. Compared to other angiosperms, one remarkable feature of the *Bh* mt genome is the frequent transfer of genetic material from the cp genome during recent *Bh* evolution. We also analyzed organellar genome evolution in general regarding genome features as well as compositional dynamics of sequence and gene structure/organization, providing clues for the understanding of the evolution of organellar genomes in plants. The cp-derived sequences including tRNAs found in angiosperm mt genomes support the conclusion that frequent gene transfer events may have begun early in the land plant lineage.

## Introduction

Plastid and mitochondria are essential organelles in plant cells. Chloroplasts conduct photosynthesis in the presence of sunlight and mitochondria indirectly supply energy within plant cells; together they form the powerhouses of the cell. Both chloroplasts and mitochondria possess their own genomes. The chloroplast (cp) genome and mitochondrial (mt) genomes are often used for the study of plant evolution [1], [2]. From the information of all sequenced cp genomes, most of them range from 120 to 160 kb in length and have GC contents of 30 to 40%. The quadripartite organization is shared by almost all cp genomes, consisting of a large-single-copy region (LSC; 80–90 kb) and a small-single-copy region (SSC; 16–27 kb), as well as two copies of inverted repeats (IRs) of ∼20 to 28 kb in size. The gene content and structure of angiosperm cp genome is highly conserved [3], [4]. Expansion and contraction of the IR as well as gene and intron losses have been documented in a wide range of angiosperms [5], [6].

The mt genome plays crucial roles in plant development and productivity [7]. In comparison to other non-plant eukaryotes, plants have large and complex mt genomes [8], [9]. Mt genomes of seed plants are unusually large and vary in size at least in an order of magnitude. Much of these variations occur within a single family [10]. Seed plant mitochondrial genomes are characteristic for their very low mutation rate [11], frequent uptake of foreign DNA by intracellular and horizontal gene transfer [12], and dynamic structure [13]. The evolving land plants have gained new mechanisms to facilitate more frequent gene exchanges between mt and cp genomes as well as between mt and nuclear genomes, which make mt genomes increase their sizes. [14].

In the past several years, we have witnessed a dramatic increase in the number of complete organellar genomes, especially those of plants. Until now, there are 206 complete cp genomes and 47 mt genomes having been deposited in GenBank Organelle Genome Resoures. With the emergence of next-generation sequencers, new approaches for genome sequencing have been gradually applied due to their high-throughput, time-saving, and low-cost advantages. With a new gene-based strategy and combining data from the two next-generation sequence platforms, pyrosequencing (Roche GS FLX) and ligation-based sequencing (Life Technologies SOLiD), we successfully assembled cp and mt genomes of resurrection plant *B. hygrometrica (Bunge) R. Br* [15]. *B. hygrometrica* or *Bh* is a small dicotyledenous, homiochlorophyllous resurrection plant in the Gesneriaceae family, and it is widespread in China, inhabiting shallow rock crevices from humid tropical regions to arid temperate zones [16], [17]. In this study, we analyze genomic features and structures of both cp and mt genomes of *B. hygrometrica*. Through organellar genome comparison with other lower plants and angiosperms, we provide information for the better understanding of organellar genome evolution in land plants.

## Results and Discussion

### Features of *B. hygrometrica* cp genome and mt genome

**Figure 1:**
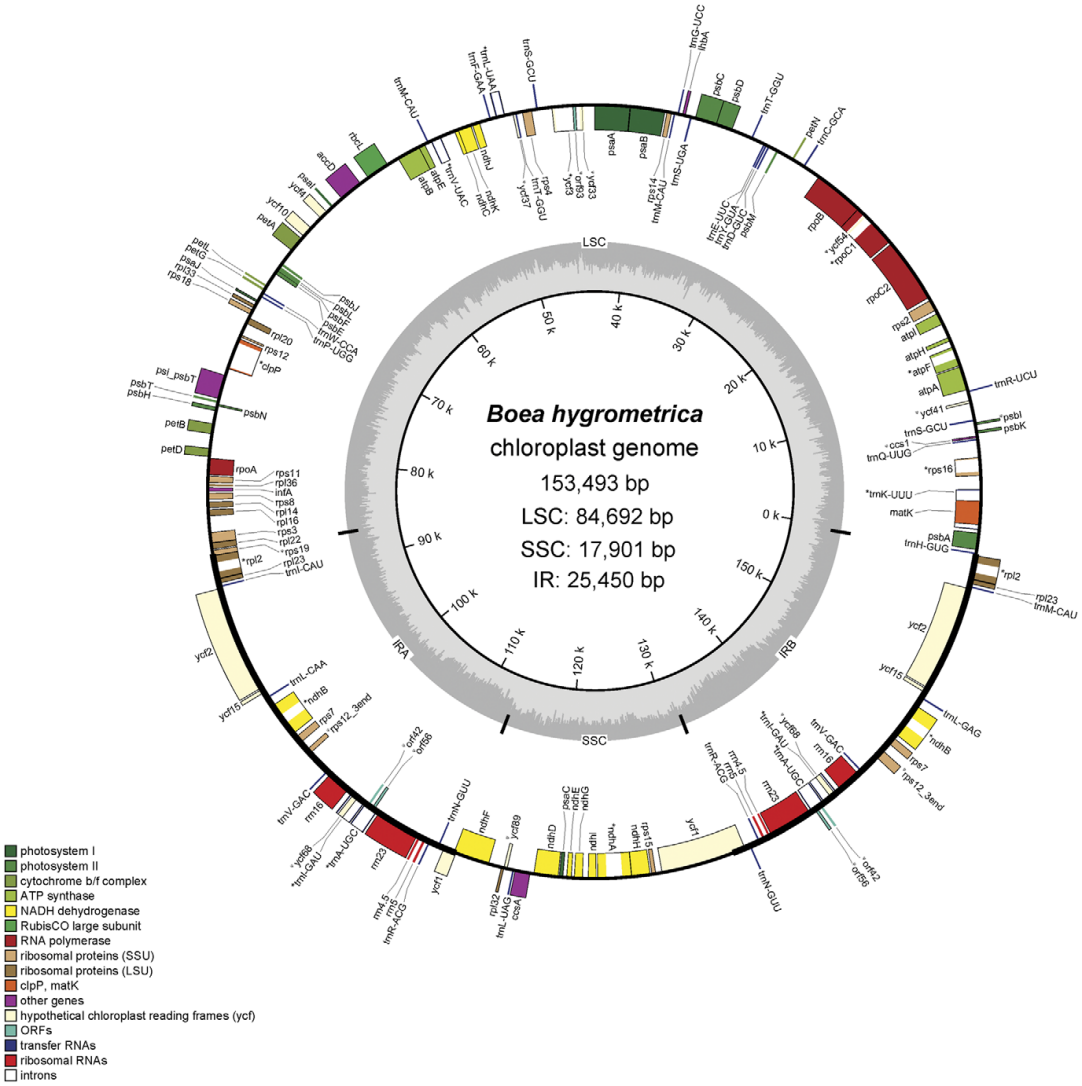
The circular-mapping chloroplast genome of *B. hygrometrica*. Features on transcriptionally clockwise and counter-clockwise strands are drawn on the inside and outside of the outer circle, respectively. Genes belonging to different groups are color-coded (Ψ, pseudogene). The genome coordinate and GC content are shown in the inner circle. The thick lines indicate inverted repeats (IRA and IRB), which separate the genome into small (SSC) and large (LSC) single copy regions. The map was drawn by using OGDRAW [47].

The *Bh* cp genome is 153,493 bp in length and has a GC content of 37.59%. Similar to those of other angiosperms [4], [18], [19], the *Bh* cp genome maps as a circular molecule with the typical quadripartite structure: a pair of IRs (25,450 bp, covering 16.6%) separated by the LSC (84,692 bp, covering 55.1%) and SSC (17,901 bp, 11.7%) regions (**Figure 1**). It encodes 147 predicted functional genes and 19 of them are duplicated in the IR regions. Among these 147 genes, we identified 103, 36, and 8 protein-coding, tRNA, and rRNA genes, respectively (**Table 1** **and** **Table 2**). 38% of the genome is non-coding, including introns, intergenic spacers, and pseudogenes. All the rRNA genes (*rrn16*, *rrn23*, *rrn5* and *rrn4.5*) and 7 tRNA (*trnI-CAU*, *trnL-CAA*, *trnV-GAC*, *trnI-GAU*, *trnA-UGC*, *trnR-ACG*, and *trnN-GUU*) genes are located in IR regions. Similar to other dicot species, *Bh* has two genes (*rps19* and *trnH*) located in the position of IR/LSC junctions. This is different in monocots, such as rice and maize, whose cpDNAs have a fully duplicated *rps19* gene in the IR/LSC junctions [18]. The average length of intergenic regions is 385 bp, varying from 1 to 2,221 bp. There are 4 cases of overlapping genes (*psbD-psbC*, *ndhK-ndhC*, *atpE-atpB*, and *ycf1-ndhF*), resulting in an average coding density (including conserved genes, unique ORFs and introns) of 1/1,058 bp. The cp genome has 19 intron-containing genes with 12 in protein-coding genes and 7 in tRNA genes. In terms of size, gene content, and intron composition, *Bh* cpDNA closely is mapped to *Olea europaea* cpDNA (155,872 bp, GC 37%) [20] among all angiosperms. Sequence alignment shows that 93% (142,189 bp) of the *Bh* cp genome sequence are covered by that of *O. europaea* with 95.4% identity (**Figure S1**). 36 tRNAs are detected, enabling *B. hygrometrica* cp genome to decode all 61 codons. All of 3 stop codons are present with UAA being the most frequently used (UAA 40%, UAG 33% and UGA 27%) (**Table S1**). Phylogenetic analyses, which were constructed by 63 protein-coding sequences from 12 cp genomes (one green algae as outgroup, one land and 10 seed plants) (**Table S2**), indicates that the phylogenetic position of *B. hygrometrica* is closer and older than *V. vinifera* among the analyzed dicots (**Figure S2**).

**Table 1:** General features of *B. hygrometrica* cp and mt genomes.

Feature	Chloroplast	Mitochondrion
Genome size (bp)	153,493	510,519
GC content (%)	37.59	43.27
Coding sequences (%)^a^	72	12.19
Gene content (%)^b^	61.21	7.89
No. of protein-coding gene	103	33
No. of intron	19	23
No. of tRNA genes	36	28
No. of rRNA operons	8	4
Repeat sequence (%)^c^	2.36	1.45
Chloroplast-derived (%)	\	10.52

a Conserved genes, unique ORFs, introns, and intron ORFs are considered as coding sequences.

b Unique ORFs and intron ORFs are not taken into account.

c Predicted by using RepeatMasker Web Server.

**Table 2:** Gene content of *B. hygrometrica* cp and mt genomes.

**cpDNA**	**Photosystem I**	*psaA,psaB,psaC,psaI,psaJ*
	**Photosystem II**	*psbA,psbB,psbC,psbD,psbE,psbF,psbH,psbI,psbJ,psbK,psbL,psbM,psbN,psbT,psi_psbT*
	**Cytochrome b/f complex**	*petA,petB,petD,petG,petL,petN*
	**ATP synthase**	*atpA,atpB,atpE,atpF,atpH,atpI*
	**NADH dehydrogenase**	*ndhA,ndhB,ndhC,ndhD,ndhE,ndhF,ndhG,ndhH,ndhI,ndhJ,ndhK*
	**RubisCO large subunit**	*rbcL*
	**RNA polymerase**	*rpoA,rpoB,rpoC1,rpoC2*
	**Ribosomal proteins (SSU)**	*rps2,rps3,rps4,rps7(×2),rps8,rps11,rps12,rps12_3end(×2),rps14,rps15,rps16,rps18,rps19*
	**Ribosomal proteins (LSU)**	*rpl2(×2),rpl14,rpl16,rpl20,rpl22,rpl23(×2),rpl32,rpl33,rpl36*
	**Other genes**	*clpP,matK,accD,ccs1,ccsA,infA,lhbA,cemA*
	**hypothetical chloplast reading frames**	*ycf1(×2),ycf2(×2),ycf3,ycf4,ycf10,ycf15(×2),ycf33,ycf37,ycf41,ycf54,ycf68(×2),ycf89*
	**ORFs**	*orf42(×2),orf56(×2),orf93*
	**Transfer RNAs**	*trnA-UGC(×2),trnC-GCA,trnD-GUC,trnE-UUC,trnF-GAA,trnG-UCC,trnH-GUG,trnI-CAU,trnI-GAU(×2),trnK-UUU,trnL-CAA,trnL-GAG,trnL-UAA,trnL-UAG,trnM-CAU(×3),trnN-GUU(×2),trnP-UGG,trnQ-UUG,trnR-ACG(×2),trnR-UCU,trnS-GCU(×2),trnS-UGA,trnT-GGU(×2),trnV-GAC(×2),trnV-UAC,trnW-CCA,trnY-GUA*
	**Ribosomal RNAs**	*rrn4.5(×2),rrn5(×2),rrn16(×2),rrn2(×2)*
**mtDNA**	**Genes of Mitochondrial Origin**	
	**Complex I (NADH dehydrogenase)**	*nad1,nad2,nad3,nad4,nad4L,nad5,nad6,nad7,nad9*
	**Complex II (succinate dehydrogenase)**	*sdh3*
	**complex III (ubichinol cytochrome c reductase)**	*Cob*
	**Complex IV (cytochrome c oxidase)**	*cox1,cox2,cox3*
	**Complex V (ATP synthase)**	*atp1,atp4,atp6,atp8,atp9*
	**Cytochrome c biogenesis**	*ccmB,ccmC,ccmFc,ccmFn*
	**Ribosomal proteins (SSU)**	*rps3,rps4,rps7,rps12,rps13*
	**Ribosomal proteins (LSU)**	*rpl16*
	**Maturases**	*matR*
	**Other genes** a	*BohyM-orf1,BohyM-orf2,BohyM-orf3*
	**Transfer RNAs**	*trnC-GCA, trnE-UUC(×2), trnG-GCC,trnI-CAU,trnK-UUU,trnL-UAA,trnM-CAU(×2), trnP-UGG, trnS-GCU,trnS-UGA,trnT-UGU, trnY-GUA*
	**Ribosomal RNAs**	*rrn5(×2),rrn18,rrn26*
	**Genes of Chloroplast Origin**	
	**Genes with intact ORFs**	*atpA,atpI,ndhB,petD,petN,psaJ,psbC,psbM,rpl20,rpl33,rpl36,rpoB,rps14,rps18,rps2,rps7,rrn16,rrn4.5,rrn5,ycf15*
	**Pseudogenes** b	*accD(×2),atpA,atpE,atpF(×2),clpP,lhbA,matK(×2),ndhC,ndhJ,ndhK,orf42,orf56,petB,psaA,psaB(×3),psbD,psi_psbT,rpl16,rpl2,rpl22,rpoA,rpoC1(×2),rpoC2(×5),rps11,rps12_3end(×2),rps16,rps3(×3),rrn23(×2),ycf2(×2),ycf41,ycf54,trnE-UUC,trnF-GAA,trnI-GAU,trnI-GAU,trnL-UAA,trnP-UGG*
	**Transfer RNAs**	*trnD-GUC,trnF-GAA(×2),trnH-GUG,trnL-CAA,trnM-CAU(×2),trnN-GUU,trnQ-UUG,trnR-ACG,trnS-GGA,trnV-GAC,trnW-CC(×2)*

a Genes that are conserve among other function unknown mitochondrial genes or ORFs.

b Genes that are transferred to mtDNA from cpDNA in fragments.

**Figure 2:**
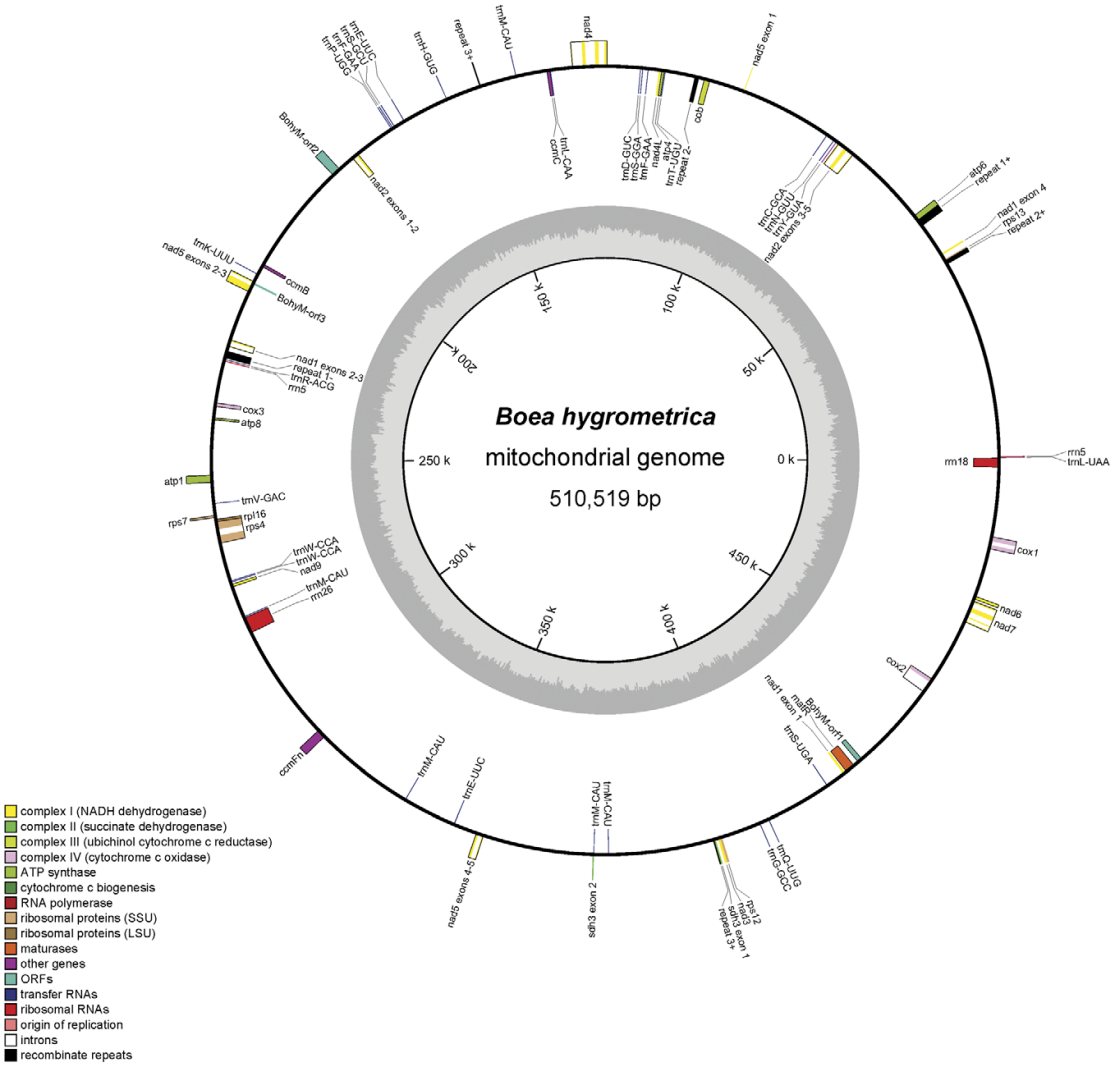
The circular-mapping mitochondrial genome of *B. hygrometrica*. Features on transcriptionally clockwise and counter-clockwise strands are drawn on the inside and outside of the outer circle, respectively. The genome coordinate and GC content are shown in the inner circle. Genes belonging to different groups are color-coded. The map was drawn by using OGDRAW [47].

The *Bh* master mt genome is assembled into a circular molecule of 510,519 bp in length (**Figure 2**) and has an average GC content of 43.27%. This size is bigger than the mt genome of *A. thaliana* (366,924 bp) [21], but smaller than *Vitis vinifera* (773,279 bp) [12] among dicots. With only 12% of coding sequences, the largest part (88%) of this genome is non-coding, containing 1.45% repeat and 10.52% cp-derived sequences (**Table 1**). The mt genome has 65 genes, including 33 protein-coding, 4 rRNA, and 28 tRNA genes, and 10 genes have exon-intron structure. Similar to other angiosperms, the *Bh* mitochondrion uses the canonical genetic code. All 61 codons are present in mt genome, and UAA (46%) is the most frequently-used stop codon (**Table S3**). The known 33 protein-coding genes in mt genome are similar to other published angiosperm mt genomes (**Table 2**), such as 9 subunits of NADH dehydrogenase (complex I), 5 subunits of ATP synthase (Complex V), and 3 subunits of cytochrome c oxidase (complex IV). Compared to other angiosperms, we observed that there is one *sdh3* and no *sdh4* in *Bh* mt genome. There are 3 copies of *sdh3* in mt genome of *Nicotiana tabacum* [22], and both *sdh3* and *sdh4* are present in that of *V. vinifera* [12]. The *Bh cox1* has an intron/exon structure that is unlike other higher seed plants (**Table S4**). There are two 5S rRNA (*rrn5*) copies and one copy of rrn5 from its cp genome. The best sequence alignment score belongs to *V. vinifera* mt genome, with 23% (119,377 bp) of *Bh* mt genome being alignable to that of *V. vinifera* with 94.2% identity (**Figure S3**).

Plant cells often contain multiple clones or copies of cp and genomes, and thus the organellar genomes can be regarded as a population with genetic heterogeneity [4]. Polymorphic sites can be detected by aligning thousands of high quality reads to assembling of the cp or mt genome [23]. Our SNP analysis shows that there is no intervarietal SNPs (intraSNPs) found in *Bh* cp genome. However, we identified 729 SNPs in *Bh* mt genome (**Table S5**) and SNPs in mt genome occurred at a rate of 1 in 700 bp. We only detected 9 SNPs in gene regions with 2, 1, 1, and 5 in *rrn5*, *rrn26*, *trnM-CAU*, and *rrn18*, respectively (**Table 3**). There are no intraSNPs detected in known protein-coding gene regions. The intraSNPs have been demonstrated to be present in both cp and mt genomes of rice [23], [24]. As an indicator for the heterogeneous nature of cp and mt populations, the intravarietal polymorphisms provide us useful markers for the future genetic studies on *B. hygrometrica*.

**Table 3:** Intravarietal polymorphisms (IntraSNPs) in the gene regions of *B. hygrometrica* mt genome.

Gene	Position	SNP	Read Coverage
*rrn5*	234508	C/T	273/212
*rrn5*	234560	G/A	320/152
*rrn26*	289003	A/G	366/86
*trnM-CAU*	339634	A/T	415/868
*rrn18*	509408	G/A	193/80
*rrn18*	509565	C/T	191/39
*rrn18*	509941	G/T	107/24
*rrn18*	509978	T/C	148/90
*rrn18*	510096	T/G	182/57

### Structural dynamics of *Bh* mitochondrial genome in ontogeny

All previous studies on complete sequencing of flowering plant mt genomes are based on the master circle (MC) hypothesis [21], [22], [25]–[28]. Arrieta-Montiel et al. reported on the structural dynamics of the common bean mt genome [29]. The analysis of 10 recombinant clones supports existence of the MC molecule in wheat mt genome [7]. However, the result of field-inversion electrophoresis suggests that *Physcombitrella* mt genome does not consist of a multipartite structure, as seen in angiosperms [30]. As mitochondrial gene orders are significantly different between lower plants and higher flowering plants, the multipartite structures as seen among angiosperms may originate during the evolution of pteridophytes or seed plants [9], [30].

**Figure 3:**
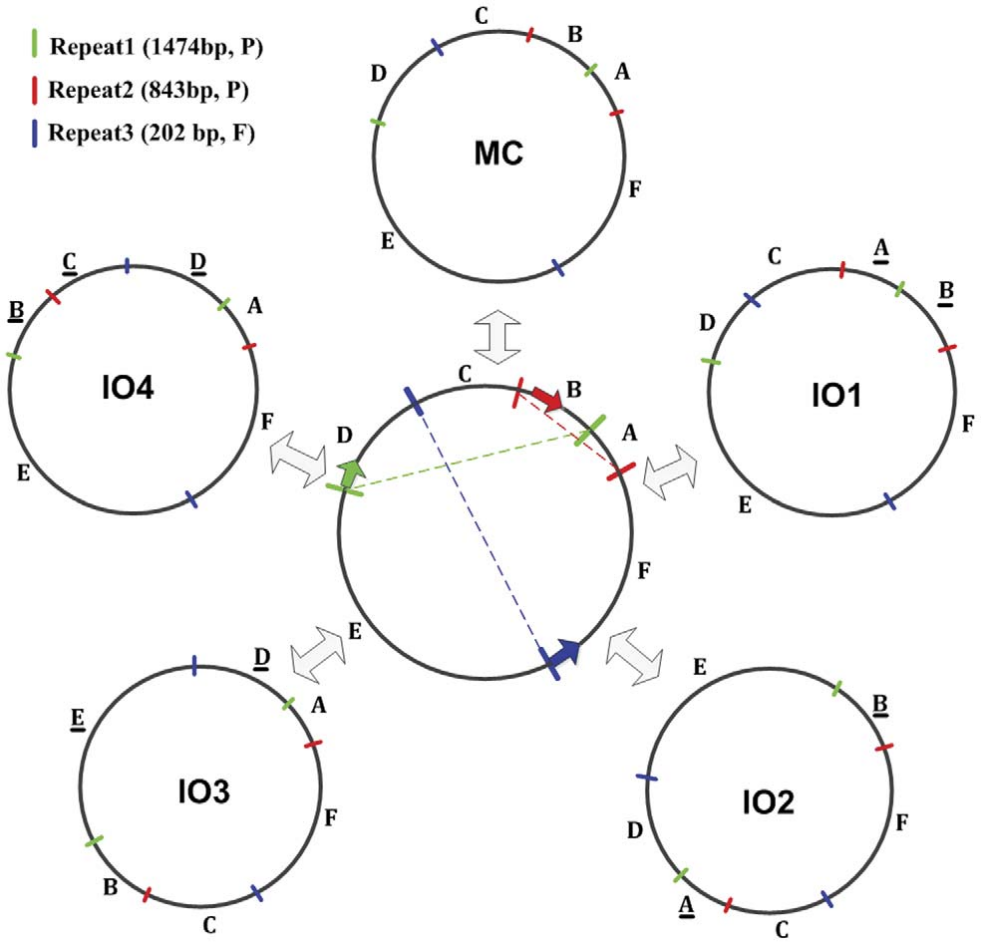
Production of various molecular structures from the MC molecule by intra-molecular recombination between 3 different repeat pairs. A, B, C, D, E, and F are 6 segments separated by repeat pairs. Underlined segments are the negative strand as compared to the MC molecule. Three repeat pairs are shown in different colors. The keys show repeat name, length, and matching direction. F and P stand for forward and palindromic matches, respectively.

Repeat prediction by REPuter shows that there are 14 repeat pairs in *Bh* mt genome (**Table 4**). Since not all the repeats are involved in recombination, from the mt assembly [15] we detected 3 repeat-specific contigs that are candidates for the recombination among the MC and other isomeric (IO) and subgenomic molecules, which have been confirmed by SOLiD sequencing [15]. Those 3 repeat contigs are the repeat pairs of two palindromic matches (1,474 bp and 843 bp) and one forward match (222 bp). By aligning all SOLiD long mate-pair reads to both ends of the repeats, we constructed the MC and 4 isomeric molecules (**Figure 3**). These subgenomic molecules are not discussed further because we have yet to find significant differences among the sequence reads.

**Table 4:** Repeat pairs predicted based on REPuter in *B. hygrometrica* mt genome.

Repeat length(bp)	Start Position	Match direction*	Repeat length (bp)	Start Position	E-value
**1474**	**51709**	**P**	**1474**	**232397**	**0.00E+00**
**843**	**42273**	**P**	**843**	**108092**	**0.00E+00**
**222**	**153742**	**F**	**222**	**405380**	**3.56E-118**
202	44767	F	202	395750	1.77E-111
180	449174	F	180	504438	4.53E-93
165	156474	P	165	272697	4.08E-84
131	25864	P	131	291976	3.89E-66
119	449235	F	119	504499	5.92E-59
116	42870	P	116	246903	3.70E-57
116	108222	F	116	246903	3.70E-57
109	300367	F	109	308910	1.74E-55
120	403184	P	120	496901	3.15E-55
102	126580	P	102	462677	1.32E-44
101	521	F	101	173834	5.13E-44

Note: the 3 recombination of repeat pairs for mt genomes are shown in bold.

*F and P stand for forward and palindromic matches, respectively.

The length of recombinant repeats (222 bp, 843 bp, and 1,474 bp) of *Bh* is different from that of *V. radiata*, and demonstrates the recombination across short mt repeats (38–297 bp) [31]. The longer repeats are reminiscent of those found in other angiosperm mitochondrial genomes, which are involved in mt genome rearrangements and can result in stoichiometric shifting of subgenomic mt genome topologies, occasionally beyond detection level for one (or more) of alternative DNA topologies [29], [32]. The 3 recombinant repeats are located in gene-rich regions and split mt genome into 6 segments. Each segment has some essential conserved genes, such as *nad1 e4* and *rps13* in segment A, and *atp6*, *nad2 e3-5* and *coxb* in segment B. From this point, it is possible that the mt genome of *Bh* do not have subgenomic molecules. There are 4 genes (*nad1*, *nad2*, *nad5* and *sdh3*) with exon-intron structure separated by the recombinant repeats (**Table 5**). The *nad1* gene in MC molecule is cross-strand gene with exon 4 in positive strand and exon 1–3 in negative strand. Recombination involving introns might lead to rearranged molecules without loss of essential genes [30]. In all rearranged 4 isomeric molecules, the Trans-splicing genes are different, and the IO3 molecule has all 4 Trans-splicing genes. Trans-splicing status of group II intron widely distributes in the mt genome of higher plant [33]–[35]. We compared gene structures of 15 mt genomes from lower to higher plants, and found 3 conserved genes (*nad1*, *nad2*, and *nad5*) as well as other higher plants contain trans-splicing intron (**Table 6**), while there is no intron in those genes of *Chara vulgaris*. The 3 genes structure supported the multipartite structures formed by multiple recombination may arise with the earliest tracheophytes [30], [32], and can be a molecular signature of plant evolution.

**Table 5:** The segments and organization of multipartite structures in *B. hygrometrica* mt genome.

Mt segment	Start position	End position	Length (bp)	Exon of cross-strand gene
**A**	43116	51708	8592	*nad1 e4*
**B**	53183	108091	54908	*nad2 e3-5; nad5 e1*
**C**	108935	153741	44806	
**D**	153964	232396	78432	*nad2 e1-2; nad5 e2-3; nad1 e2-3*
**E**	233871	405379	171508	*nad5 e4-5; sdh3 e2*
**F**	405602	42272	147189	*sdh3 e1; nad1 e1*

Molecule	Organization	Cross-strand gene
**MC**	ABCDEF	*nad1*
**IOS1**	BACDEF	*nad2; nad5*
**IOS2**	BEDACF	*nad2; nad5*
**IOS3**	ADEBCF	*nad1; nad2; nad5; sdh3*
**IOS4**	ADCBEF	*nad1; nad5*

Note: segments or exons in negative strand are underlined.

**Table 6:** Structural comparison of *nad1*, *nad2*, and *nad5* genes in plant mt genomes.

Species	*nad1*	*nad2*	*nad5*
*Chara vulgaris*	−*	+*	+*
*Marchantia polymorpha*	+*	++	++
*Megaceros aenigmaticus*	++++	+++	++++
*Cycas taitungensis*	+−−−−	−−+++	+++++
*Triticum aestivum*	−−−−+	++−−−	+++++
*Oryza sativa*	+−−−−	+++−−	++−−−
*Sorghum bicolor*	+−−+−	−−+++	++−++
*Tripsacum dactyloides*	−−−−+	++−−−	+++++
*Zea mays*	++++−	−−+++	++−−−
*Beta vulgaris*	−−−+−	++−−−	+++−−
*Brassica napus*	−++++	−−+++	++−−−
*Arabidopsis thaliana*	−−−−−	−−−−−	−−−−−
*Nicotiana tabacum*	+−−−−	−−+++	++−++
*Vitis vinifera*	+++−−	+++++	−−−−−
*Boea hygrometrica*	−−−++	−−−−−	+++++

Note: “+” or “−” shows exons located in positive or negative strands, respectively.

“*” indicates genes that lack exon-intron structures.

The number of “+” or “−” strands for the number of exons in a gene.

### Comparative analysis of cp genome organization

We compared 12 cp genomes ranging from green algae to angiosperm (**Table S6**). The GC contents of cp genome are lowest in lower plants (Charophyta and Bryophyta) but highest in Cycads. Monocots seem to have slightly higher GC contents than dicots among their cp genomes. The genome size and structure of cp genomes are also different in those cp genomes. For example, *C. vulgaris* (184,933 bp; Charophyta) has the largest genome while the smallest genome is found in lower plant *Marchantia polymorpha* (121,024 bp). The genome size of angiosperms is more stable than lower plants with dicots larger than monocots. Compared to lower pants, the most variable portions of angiosperm cp genomes are percentages of IRs (34% in *A. thaliana*) and LSC (54.5% in *A. thaliana*) regions. This is the result of IR expansion into the LSC region from green algae to angiosperm [4].

The cp genome contains genes that encode structural and functional components of the organelle. Although some genes and gene clusters are well conserved among all plants, the overall structure of cp genomes show remarkable differences (**Table S6**). First, there are 63 core protein-coding genes, shared among all plants, whereas there are 3 additional core genes (*chlB*, *chlL* and *ycf12*) only found in the lower plant lineage (Charophyta, Bryophyta, and Cycads). The 63 core cp genes are involved in photosynthesis, energy metabolism, and other housekeeping functions. Second, there are 10 genes (*psaM*, *rpl5*, *rpl12*, *rpl19*, *tufA*, *ycf20*, *ycf62*, *ycf66*, *odpB*, and *ftsH*) are unique to green algae. All of them reside in the LSC region except *ycf20* gene that is duplicated in IR regions. Compared to seed plants, there is only one gene, ribosomal protein L21 (*rpl21*) is conserved in both green algae and liverwort. Third, all four ribosomal RNA genes (*rrn4.5*, *rrn5*, *rrn16*, and *rrn23*) have 2 copies in IR regions except the 2 copies of *rrn4.5* that is lost in Charophyta. Fourth, gene loss and transfer to the nucleus is a common feature of cp genomes [36]. We detected 3 genes (*petL*, *petN*, and *ycf3*) that are lost at the base of the Bryophyta lineage and 2 genes (*accD* and *ycf2*) are lost in monocots as compared to dicots. There are also some species-specific gene lost events, such as *psaJ* in *O. sativa* and *nadJ*-*ccsA* lost in *O. europaea* [20]. The unique loss of *psbZ* in LSC region testifies the convergent evolution of *B. hygrometrica* and *O. europaea*.

The order of cp genes in plants is not constant, changing among different regions of the genome as large gene clustering become rare. Among 63 core protein-coding genes, 50 are always reside in LSC region, 5 (*psaC*, *ndhD*, *ndhE*, *ndhG*, and *ndhI*) in SSC region, and 8 (*ndhA*, *ndhB*, *ndhF*, *ndhH*, *rpl2*, *rpl23*, *rpl32* and *rps7*) in variable positions among 12 examined cp genomes. No conserved protein-coding genes are found constant in IR regions. These mobile genes may serve as an indication of lineage markers, since 4 of them (*ndhB*, *rpl2*, *rpl23*, and *rps7*) locate on LSC region in lower plants and migrated to IR regions in higher plants. Genes residing in the boundary of LSC/IRA or IRB/LSC are usually ribosomal proteins S12 (*rps12*) in higher plants and the position-conserved *ycf1* is more likely present in the boundary of IRA/SSC and SSC/IRB in dicots.

### Comparative analysis of plant mt genomes

The plant mt genomes are exceptionally variable in size, structure, and sequence content and the accumulation of repetitive sequences contributes the most to such variation [31]. From the feature comparison of 15 plant mt genomes (**Table 7**), we noticed that their genome sizes vary from 67,737 bp in *C. vulgaris* to 773,279 bp in *V. vinifera*. Recently, the large mt genome have been reported in *Cucurbita pepo* with 982,833 bp [10]. The GC contents of these genomes are also variable from 40 to 47*%*. There is a massive difference of coding sequences between lower and higher plants. The coding sequence in *C. vulgaris* is 90.7%, whereas it is 4.94% in *Tripsacum dactyloides*. Repeat content ranges from 1 to 41% among seed plants and are smallest in *B. hygrometrica*, composed of only 1.45% of the genome. Both large (>1,000 nt) and small (<50 nt) repeats affect recombination in seed plants [7], [31], [37]. The protein-coding genes and tRNAs in mt genomes also vary largely because of the large number of function-unknown proteins or ORFs in mt genomes and frequent plastid DNA insertions including cp tRNA genes [26], [38].

**Table 7:** Comparison of basic features among 15 mt genomes.

Scientific Name	Size (bp)	GC (%)	Coding (%)	Repeats (%)	Protein-coding genes	tRNAs	rRNAs
***Chara vulgaris***	67,737	40.9	90.7	3.2	46	27	3
***Marchantia polymorpha***	186,609	42.41	20.3	10.1	76	29	3
***Megaceros aenigmaticus***	184,908	46.01	17.9	2.6	48	18	3
***Cycas taitungensis***	414,903	46.92	10.1	15.1	39	26	3
***Triticum aestivum***	452,528	44.35	8.6	10.1	39	25	9
***Oryza sativa***	490,520	43.85	11.1	28.8	53	22	3
***Sorghum bicolor***	468,628	43.73	6.69	13.1	32	18	3
***Tripsacum dactyloides***	704,100	43.93	4.94	40.6	33	18	3
***Zea mays***	569,630	43.93	6.2	11.4	163	29	4
***Beta vulgaris***	368,801	43.86	10.3	12.5	140	26	5
***Brassica napus***	221,853	45.19	17.3	5.5	79	17	3
***Arabidopsis thaliana***	366,924	44.77	10.6	7.8	117	21	3
***Nicotiana tabacum***	430,597	44.96	9.9	10.8	156	23	4
***Vitis vinifera***	773,279	44.14	4.98	1.71	74	31	3
***Boea hygrometrica***	**510,519**	**43.27**	**12.19**	**1.45**	**33**	**28**	**4**

Note: Parts of the dataset are available from published data [53].

We also carefully examined conserved genes in different plant lineages (**Table S4**). First, there are 14 conserved core protein-coding genes shared among all lineages, including seven subunits of NADH dehydrogenase (Complex I), one subunit of ubichinol cytochrome c reductase (Complex III), three subunits of cytochrome c oxidase (Complex IV), and three subunits of ATP synthase (Complex V). All these genes play important roles either in proton movement across the inner membrance of the mitochondrion or electron transfer reactions in the respiratory chain. However, the gene structures are not conserved among them, and only two genes (*nad4* and *cox2*) have exon-intron structures in all mt genomes. For comparison, there are 9 genes (*nad3*, *nad4L*, *nad6*, *nad9*, *cob*, *cox3*, *atp1*, *atp9*, and *ccmFN*) without exon-intron structure in both seed and early land plants and with exon-intron structure at least in one lower plant. Intron structure in mt genes is common as we only detected 6 genes (*sdh3*, *sdh4*, *atp4*, *atp8*, *ccmB*, and *ccmFN*) have no introns among all plants. Second, gene loss is more frequent in dicots than monocots, as genes in cytochrome c biogenesis are lost in both *B. vulgaris* and *A. thaliana*. Three species (*Nicotiana tabacum*, *V. vinifera* and *B. hygrometrica*) gained *sdh3* as in this study. The number of ribosomal protein genes is different in various plant mt genomes. Most ribosomal proteins (23) are present in *V. vinifera* genome. Contrast to higher plants, there is no *matR* detected in liverwort and green algae in this study. However, it is reported that in mosses, Takakia and Sphagnum have part of *matR* [35], [39]. Most of mt genomes in plants have 3 ribosomal RNAs (*rrn5*, *rrn18*, and *rrn26*), but there are multiple copies found in angiosperms (such as *T. aestivum* and *B. vulgaris*). Copy number-variable mt genes are reported in wheat, rice, and maize [7]. In summary, since the gene coding fraction is much less among mt genomes as compared to cp genomes, even conserved genes are also variable in gene content, structure, and intron positioning [30].

### Plastid DNA insertions in mt genome

One of the important events in determining mt genome size in angiosperms is the frequent capture of sequences from the cp genome [10], [26], [28], [40]. A recently study demonstrates frequent DNA transfer from cp to mt genomes occur as far back as the common ancestor of the extant gymnosperms and angiosperms, about 300 MYA and the frequency of cp-derived sequence transfer is positively correlated with variations in mt genome size [41]. For instance, *B. hygrometrica* mt genome contains fragments of cp origin, ranging from 50 to 5,146 bp in length (**Table S7**). The total fraction of cp-derived sequences present in *Bh* mt genome is 53,440 bp, 10.5% of the whole mt genome. Most of these insertions are conserved, as evidenced from the observation that 45 out 80 insertions (over 50 bp) are identified in mt genomes of other plants. The average GC content between old insertions (at least found one homolog in the mt genomes of other plants) and new insertions (no homologs found in other mt genomes) is obviously different (**Figure S4**). The average GC content of old insertions (41.59%) is distinct from that of mt genomes (43.27%) as well as the average GC content of new insertions (35.32%) is close to the GC content of cp genomes (37.59%; **Table 8**). The result suggests positive correlations between the GC content of cp genomes and new insertions (coefficient value r^2^ = 0.69) and between the GC content of mt genomes and old insertions (coefficient value r^2^ = 0.64). There is a significant difference between the GC content of old and new insertions (T test: *P<0.01*). All of cp-derived sequences in lower plants are old insertion, and it is strange to see there are no new insertions from cp genomes during the evolution of *A. thaliana*. Compared to other angiosperms, the most striking feature of *Bh* mt genome is thefrequent sequence transfer from the cp genome in its recent evolution. Cp-derived sequence analyses may provide clues for the understanding functional indications of DNA insertions from cp in mt genomes.

**Table 8:** Comparison of cp-derived sequences in mt genomes among plants.

Species	*Chara*	*Marchantia*	*Cycas*	*Triticum*	*Oryza*	*Sorghum*	*Zea*	*Arabidopsis*	*Nicotiana*	*Vitis*	*Boea*
**Cp-GC (%)**	26.19	28.81	39.45	38.31	38.99	38.49	38.46	36.29	37.85	37.4	37.59
* **Mt-GC (%)**	40.9	42.41	46.92	43.93	43.85	43.73	43.93	44.77	44.96	44.14	43.27
*Mtpt No	11	16	40	57	71	49	41	30	43	51	80
*Mtpt **length (bp)**	915	1379	17608	15089	34969	33006	24859	5150	11415	68925	53440
Old mtpt	11	16	21	54	63	44	38	30	39	33	45
New mtpt	0	0	19	3	8	5	3	0	4	18	35
**Old-GC (%)**	*54.54*	52.79	45.85	44.63	40.62	41.23	44.55	46.06	46.2	40.52	41.59
**New-GC (%)**	\	\	40.75	34.49	37.72	39.47	42.62	\	34.09	34.08	35.32

*Mtpt: cp-derived sequences from chloroplast to mitochondrial genomes. Only the genus names are indicated.

We also analyzed the inserted cp genomic sequences to mt genomes. First, there are 85 cp-derived fragments in the *Bh* mt genome. Most of them, especially protein-coding sequences, have no intact gene structure or have frameshifts/indels and the fact suggests that theses cp-derived sequences are degenerated and lack functional constraints [41]. However, protein-coding genes such as ribosomal proteins and tRNAs originated from cp genomes appear still functional in *Bh* mt genome. Similar genes are also seen in other angiosperms [26], [28]. Second, it is curious to investigate the locations of cp-derived sequences in cp DNAs to see if any particular regions in cp genomes are hotspots of DNA transfer. Among 80 transferred sequence fragments in *Bh* cp genome, 45, 7, and 28 are from LSC, SSC, and IR regions, respectively. The numbers of fragments appear correlating to the lengths of LSC (84,692 bp), SSC (17,901 bp) and IRs (50,900 bp), and such correlations are also seen in other angiosperms, including maize, rice, wheat, and tobacco [41]. In conclusion, DNA transfer from cp genomes to mt genomes in angiosperms occurs randomly as it has been proposed earlier by Mastsuo et al in rice [42].

### tRNAs transfer between cp and mt genomes

To investigate whether mt genomes encodes a full set of tRNAs species necessary for protein synthesis in the organelle, we identified 28 tRNA genes from the complete *Bh* assembly based on tRNA structures and realized that all 61 codons are used by *Bh* mitochondria (**Table S3**). However, the tRNA genes encoded by the mt genome alone are not sufficient to decode all codons; for instance, *trnA* is missing in *Bh*, and it suggests that the role of the missing tRNA is supplied by either cp or nuclear genomes [22], [26], [41]. tRNAs originated from plastids are called cp-derived tRNAs and their counterparts are native mt tRNAs. Half of the 28 mt tRNAs in *B.hygrometrica* are identified as cp-derived tRNAs (**Table S8**) and 19 amino acids are encoded by only one codon except for leucine (UAA and CAA) and serine (GCU, UGA, and GGA).

**Figure 4:**
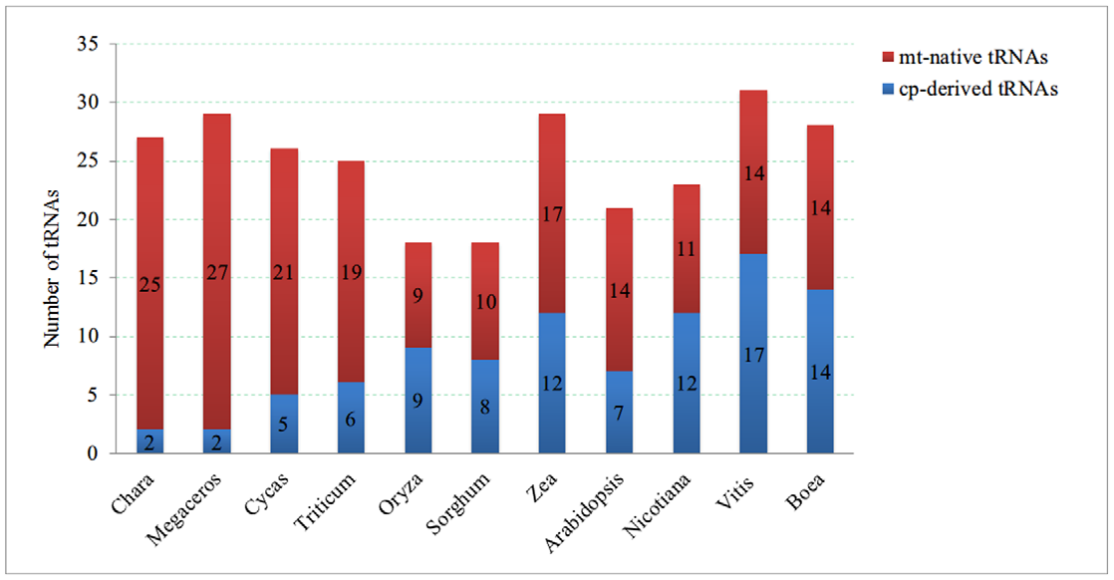
Distribution of tRNA genes in 15 plant mt genomes.

In contrast to the protein coding genes, mt tRNA genes appear constantly being transferred from cp genomes during the evolution of angiosperms (**Figure 4** **and Table S8**), and the proportion of cp-derived tRNAs in mt genomes increases from 8% in Charophyta to 55% in dicotyledonous plants. There are 17 cp-derived tRNAs and 14 mt native tRNAs in mt genome of *V. vinifera*, which has the most cp-derived tRNAs among dicotyledonous plants. Seven mt-native tRNA genes (*trnD-GUC*, *trnE-UUC*, *trnI-CAU*, *trnK-UUU*, *trnM-CAU*, *trnS-GCU*, *trnS-UGA* and *trnW-GUA*) and one cp-derived tRNA gene (*trnF-GAA*) are common to all 15 species. Compared to the mt native tRNA genes in lower plants, there are three tRNA genes (*trnH-GUG*, *trnN-GUU*, and *trnW-GUA*) integrated as part of large cp genomic fragments into mt genomes among angiosperms [26]. This indicates that frequent DNA transfer from cp to mt genomes occur as far back as the emergence of seed plants [41]. We detected two different tRNAs transfer events in seed plants. One is *trnC-GCA* transfer in monocots and the other involves two (*trnD-GUC* and *trnQ-UUG*) gene transfer events in dicots. Cp-derived tRNA genes replace their mt counterparts were identified in all sequenced angiosperms, even in gymnosperm *Cyas* mtDNA. But these replacement not occurred in *Marchantia*, *Reclinomonas*, *Cyanidioschyzon*, *Nephroselmis*, *Chara*, and *Physcomitrella* [43].The mt-native tRNA gene (*trnG-GCC*) had all been lost in monocots and six mt-native genes (*trnA-UGC*, *trnG-UCC*, *trnL-UAG*, *trnR-UCU*, *trnR-ACG*, and *trnT-GGU*) are mostly lost in all angiosperms.

### Gene gain and loss in plant organelle genome

**Figure 5:**
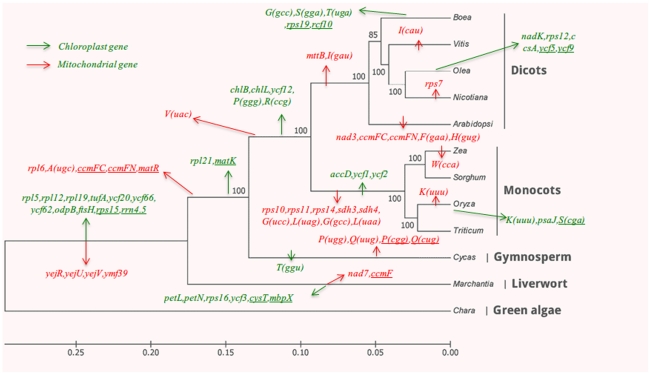
Phylogenetic distribution of gene gain and loss from chloroplast and mitochondrial genomes in plant lineages. The genes that are gained independently in different lineages are underlined. Genes of chloroplast and mitochondrial origins are indicated with green and red, respectively. Phylogenetic relationships of the plants are determined based on the conserved genes of chloroplast genomes described in Figure S2.

Starting from *Bh* organellar genomes, we have analyzed in a systematic way representative cp and mt genomes of various lineages and our results provide information for a better understanding of organellar genome evolution and function. Sequence-based phylogenetic analysis clearly supports the conclusion that *Bh* is much close to *V. vinifera*. Structural dynamics of *Bh* mt genome suggest that the multipartite structures may have started during the evolution of seed plants [30]. However, mechanisms for rapid mt genome rearrangement and expansion among plant lineages remain enigmatic. Based on eleven known cp and mt genomes of different lineages, we showed a strong relationship between the changing organellar genomes among angiosperms, and some of the lineage-associated gene gain and loss may provide excellent markers for phylogenetic studies (**Figure 5**). For instance, there are 9 cp and 4 mt genes lost during the evolution from green algae to lower land plants. It seems that monocots have a faster rate of evolution than dicots in organellar genomes in our study, because 3 cp and 9 mt genes are lost in monocots and only 2 mt genes are lost in dicots. In additional, gene structures and positioning of cp and mt genomes are also very informative for the understanding of land plant evolution. In agreement with the results of several previous studies, most of the transferred angiosperm sequences from cp to mt genomes become degenerated and are regarded as junk sequences, whereas some of the cp-derived tRNAs are still functional in mt genomes [26], [28], [41]. As more plant organellar genome sequences become available, the evolution of plant organellar genomes will unveil its details and mechanisms.

## Materials and Methods

### Genome sequencing and assembly

We developed an efficient procedure for *Bh* organellar genome sequencing and assembly using whole genome data from 454 GS FLX sequencing platform [15]. Briefly, we collected fresh leaves and extracted genomic DNA for 454 GS FLX sequencing (see manuals of GS FLX Titanium for detail). In order to validate genome assembly and to make sure for the assembly of the master circle or MC, we construed two mate-pair libraries (2×50 bp) for SOLiD 4.0 sequencing platform with insert sizes of 1–2 kb and 3–4 kb by following the SOLiD Library Preparation Guide. The method for assembling organellar genome was based on correlation between contig read depth and copy number in the genome [44]. We first filtered cp reads from the raw data according known plant cp genome sequences and then assembled the “clean” read into cp genome into the major segments: large-single-copy (SSC), small-single-copy (SSC) and inverted repeats (IRs) regions. The mt genome assembly is more complicated than that of cp genomes. We filtered the contigs including mt conserve genes (such as NADH dehydrogenase and succinate dehydrogenase) and removed the contamination of cp sequences. The gene-based method for assembling mt genome has been reported earlier [7]. Mapping all the SOLiD mate-pair reads to mt contigs with Bioscope, we obtained the major contig relationship map in the repeat regions to assemble the MC.

### Genome annotation

The cp genome was annotated by using the program DOGMA (Dual Organellar GenoMe Annotator) [45] coupled with manual corrections for start and stop codons. Protein-coding genes are identified by using the plastid/bacterial genetic code. Codon usage is predicted by using CodonW (http://codonw.sourceforge.net/). We construct a custom-designed amino acid database for protein-coding genes and nucleotide databases for rRNA and tRNA genes, compiled from all previously annotated plant mt genomes available at the organelle genomic biology website at NCBI (http://www.ncbi.nlm.nih.gov/genomes/ORGANELLES/organelles.html). NCBI BlastX and BlastN searches of the mt genome against the databases allow us to find protein and RNA genes, respectively. All BlastN and BlastX searches are carried out by using the default settings with e-value 1e-10. Putative RNA editing sites are inferred to create proper start and stop codons as well as to remove internal stop codons. We also used tRNAscan-SE [46] to corroborate tRNA boundaries identified by BlastN. The annotated GenBank files of the cp and mt genomes of *Bh* are used to draw gene maps using OrganellarGenome DRAW tool (OGDRAW) [47]. The maps were then examined for further comparison of gene order and content.

### Analyses on SNPs, repeats, and cp-derived sequences

We identified intra-specific SNPs in both cp and mt genomes. Using BioScope, we mapped two runs of SOLiD mate-pair reads to both cp and mt genomes (BioScope Software User Guide). We carried out repeat sequence analysis using the REPuter web-based interface (http://bibiserv.techfak.uni-bielefeld.de/reputer/) [48], including forward, palindromic, reverse and complemented repeats with a minimal length of 50 bp. Transposable elements and other repeated elements were mapped with RepeatMasker Web Server (http://www.repeatmasker.org/cgi-bin/WEBRepeatMasker) running under the cross_match search engine. Cp-derived sequences are identified with BlastN search of mt genomes against *Bh* annotated cp genomes (Identity ≥80%, E-value≤1e-5, and Length ≥50 bp). The cp-derived sequences were then aligned to all known plant mt genomes by using BlastN (Identity ≥80%, E-value≤1e-5, and Coverage ≥50%). tRNAs transferred to the mt genome were identified by aligning to all tRNAs in the cp genome of the same species by using BlastN (Identity ≥80%, E-value≤1e-5, and Coverage ≥50%).

### Phylogenetic Analysis

We compare the *Bh* cp genome with other plant organellar genomes, and use the homologous protein-coding sequences to construct phylogenetic tree. Sixty-three cp protein sequences (*psaA*, *psaB*, *psaC*, *psaI*, *psbA*, *psbB*, *psbC*, *psbD*, *psbE*, *psbF*, *psbH*, *psbI*, *psbJ*, *psbK*, *psbL*, *psbM*, *psbN*, *psbT*, *petA*, *petB*, *petD*, *petG*, *atpA*, *atpB*, *atpE*, *atpF*, *atpH*, *atpI*, *rbcL*, *rpoA*, *rpoB*, *rpoC1*, *rpoC2*, *ndhA*, *ndhB*, *ndhC*, *ndhD*, *ndhE*, *ndhF*, *ndhG*, *ndhH*, *ndhI*, *ndhJ*, *rpl2*, *rpl14*, *rpl16*, *rpl20*, *rpl22*, *rpl23*, *rpl32*, *rpl33*, *rpl36*, *rps2*, *rps3*, *rps4*, *rps7*, *rps8*, *rps11*, *rps14*, *rps18*, *clpP*, *cemA*, and *ycf4*) from 12 different organisms (**Table S2**) are aligned and concatenated into a dataset of 196,313 amino acids.

We align amino acid sequences from individual genes using MUSCLE v3.8.31 [49], remove ambiguously aligned regions in each alignment using GBLOCKS 0.91b [50], and concatenate the aligned sequences. We use maximum likelihood method and PhyML v3.0 [51] under Jones-Taylor-Thornton (JTT and gamma distribution of rates across sites with four categories) model of sequence evolution to construct phylogenetic trees. Confidence of branch points is estimated based on 100 bootstrap replications. We obtained the best tree after heuristic search with the help of Modelgenerator [52].

### Accession Numbers

The GenBank accession numbers for the sequences mentioned in this article are as follows: *Chara vulgaris*, NC_008097 and NC_005255; *Marchantia polymorpha*, NC_001319 and NC_001660; *Megaceros aenigmaticus*, NC_012651; *Cycas taitungensis*, NC_009618 and NC_010303; *Triticum aestivum*, NC_002762 and NC_007579; *Oryza sativa*, NC_001320 and NC_011033; *Sorghum bicolor*, NC_008602 and NC_008360; *Tripsacum dactyloides*, NC_008362; *Zea mays*, NC_001666 and NC_007982; *Beta vulgaris*, NC_002511; *Brassica napus*, NC_008285; *Arabidopsis thaliana*, NC_000932 and NC_001284; *Nicotiana tabacum*, NC_001879 and NC_006581; *Vitis vinifera*, NC_007957 and NC_012119; *Olea europaea*, NC_013707; *Boea hygrometrica*, JN107811 and JN107812.

## Supporting Information

Figure S1 Chloroplast genomic alignment between *Boea hygrometrica* and *Olea europaea*. Alignments with direct match are shown in red and reverse match are shown in blue. Obviously, alignments of two IR regions are indicated in blue.
(DOC)

Figure S2 Molecular phylogenetic analysis based on Maximum Likelihood method by using JTT matrix-based model (*Chara vulgaris* as outgroup). The bootstrap consensus tree inferred from 100 replicates is taken to represent the evolutionary history of selected plants. The dataset is composed of sixty-three conserved cp proteins concatenated to 14,894 positions from 12 plant cp genomes. Nodes receive over 80% bootstrap replicates are indicated at phylogenetic positions and *B. hygrometrica* is next to *Vitis vinifera* among dicots.
(DOC)

Figure S3 Mitochondrial genomic alignment between *Boea hygrometrica* and *Vitis vinifera*. Alignments with direct match are shown in red and reverse match are shown in blue.
(DOC)

Figure S4 The GC distribution between new and old cp-derived sequences in mitochondrial genome of *Boea hygrometrica*. Hits stand for the results of homologous alignments with other known mitochondrial genomes.
(DOC)

Table S1 Table Codon usage table of the chloroplast genes.
(DOC)

Table S2 cp and mt genomes of 15 plants comparison in this study.
(DOC)

Table S3 Codon usage table of the mitochondrial genes.
(DOC)

Table S4 Gene content and characteristic comparison of 15 mt genomes.
(XLS)

Table S5 Intravarietal single nucleotide polymorphisms (intraSNPs) in mt genome.
(XLS)

Table S6 Gene content and characteristic comparison of 12 cp genomes.
(XLS)

Table S7 Blast result of chloroplast-derived DNA segment in mitochondrial genome of *Boea hygrometrica*.
(XLS)

Table S8 tRNAs comparison of both cp and mt genomes among 11 pant.
(XLS)
