# Supplementary material for: The Complete Chloroplast and Mitochondrial Genome Sequences of Boea hygrometrica: Insights into the Evolution of Plant Organellar Genomes
Source: PLoS One. 2012 Jan 23;7(1):e30531. doi: 10.1371/journal.pone.0030531 (PMC3264610; doi:10.1371/journal.pone.0030531)
Supplement: Figure S1 — Chloroplast genomic alignment between Boea hygrometrica and Olea europaea. Alignments with direct match are shown in red and reverse match are shown in blue. Obviously, alignments of two IR regions are indicated in blue. (DOC) [file pone.0030531.s001.doc]

**S1.** Chloroplast genomic alignment between *Boea hygrometrica* and *Olea europaea*. Alignments with direct match are shown in red and reverse match are shown in blue. Obviously, alignments of two IR regions are indicated in blue.


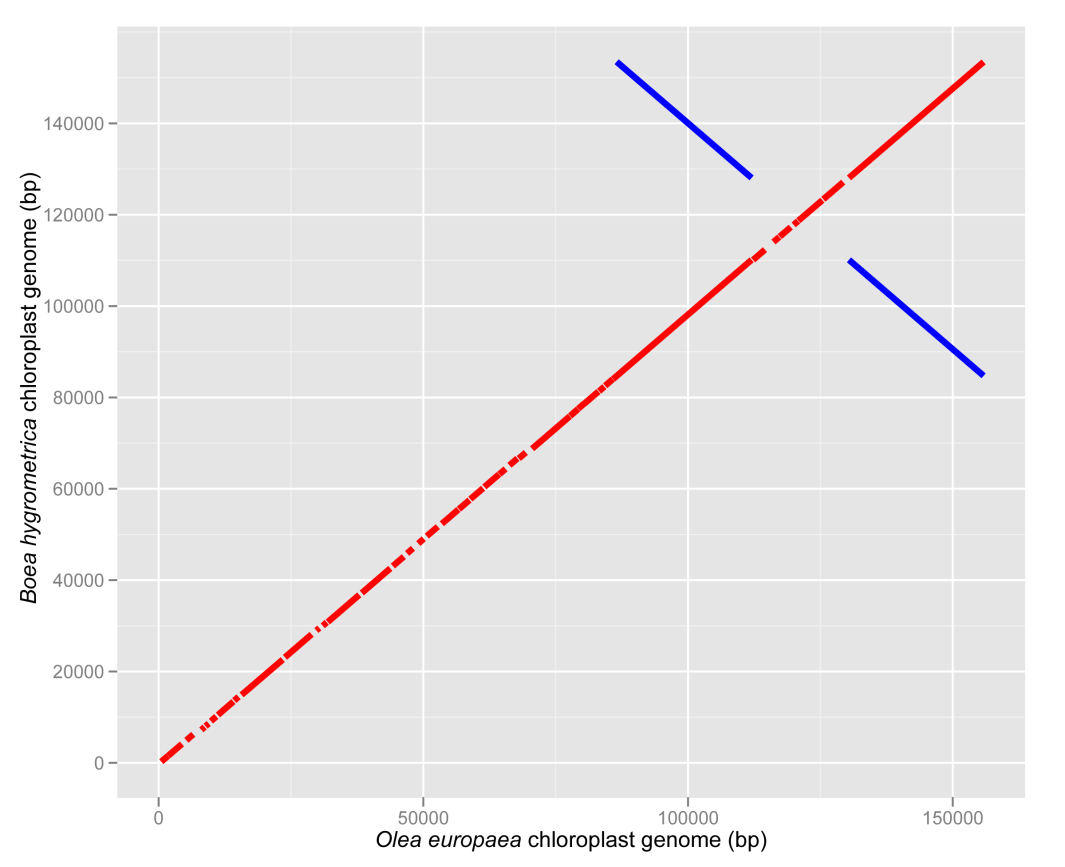


**Fig S1**
