# Supplementary material for: The Complete Chloroplast and Mitochondrial Genome Sequences of Boea hygrometrica: Insights into the Evolution of Plant Organellar Genomes
Source: PLoS One. 2012 Jan 23;7(1):e30531. doi: 10.1371/journal.pone.0030531 (PMC3264610; doi:10.1371/journal.pone.0030531)
Supplement: Figure S2 — Molecular phylogenetic analysis based on Maximum Likelihood method by using JTT matrix-based model (Chara vulgaris as outgroup). The bootstrap consensus tree inferred from 100 replicates is taken to represent the evolutionary history of selected plants. The dataset is composed of sixty-three conserved cp proteins concatenated to 14,894 positions from 12 plant cp genomes. Nodes receive over 80% bootstrap replicates are indicated at phylogenetic positions and B. hygrometrica is next to Vitis vinifera among dicots. (DOC) [file pone.0030531.s002.doc]

**S2.** Molecular phylogenetic analysis based on Maximum Likelihood method by using JTT matrix-based model (*Chara vulgaris* as outgroup). The bootstrap consensus tree inferred from 100 replicates is taken to represent the evolutionary history of selected plants. The dataset is composed of sixty-three conserved cp proteins concatenated to 14,894 positions from 12 plant cp genomes. Nodes receive over 80% bootstrap replicates are indicated at phylogenetic positions and *B. hygrometrica* is next to *Vitis vinifera* among dicots.

**
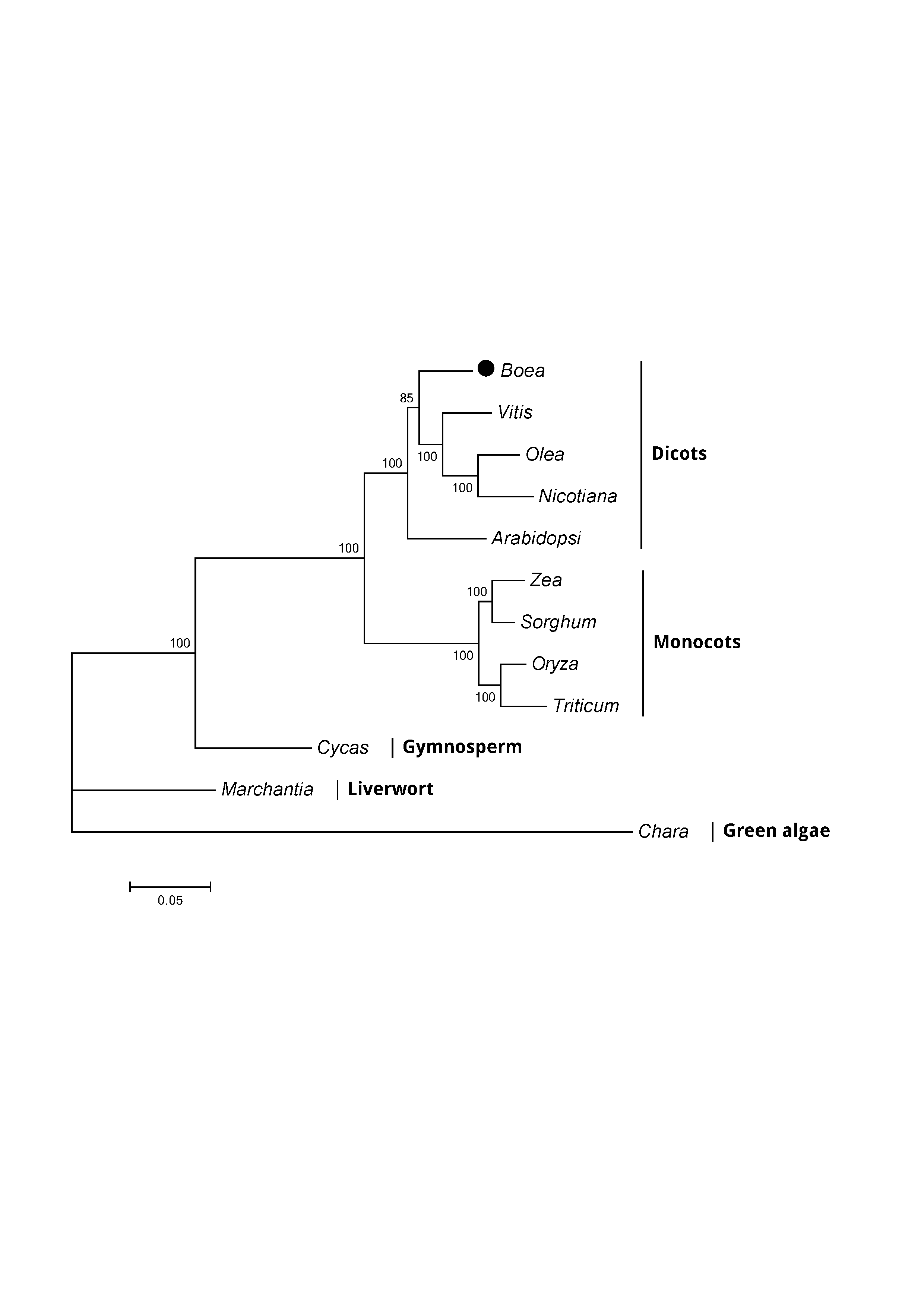
**

**Fig S2**
