# Supplementary material for: The Complete Chloroplast and Mitochondrial Genome Sequences of Boea hygrometrica: Insights into the Evolution of Plant Organellar Genomes
Source: PLoS One. 2012 Jan 23;7(1):e30531. doi: 10.1371/journal.pone.0030531 (PMC3264610; doi:10.1371/journal.pone.0030531)
Supplement: Figure S3 — Mitochondrial genomic alignment between Boea hygrometrica and Vitis vinifera. Alignments with direct match are shown in red and reverse match are shown in blue. (DOC) [file pone.0030531.s003.doc]

**S3.** Mitochondrial genomic alignment between *Boea hygrometrica* and *Vitis vinifera*. Alignments with direct match are shown in red and reverse match are shown in blue.


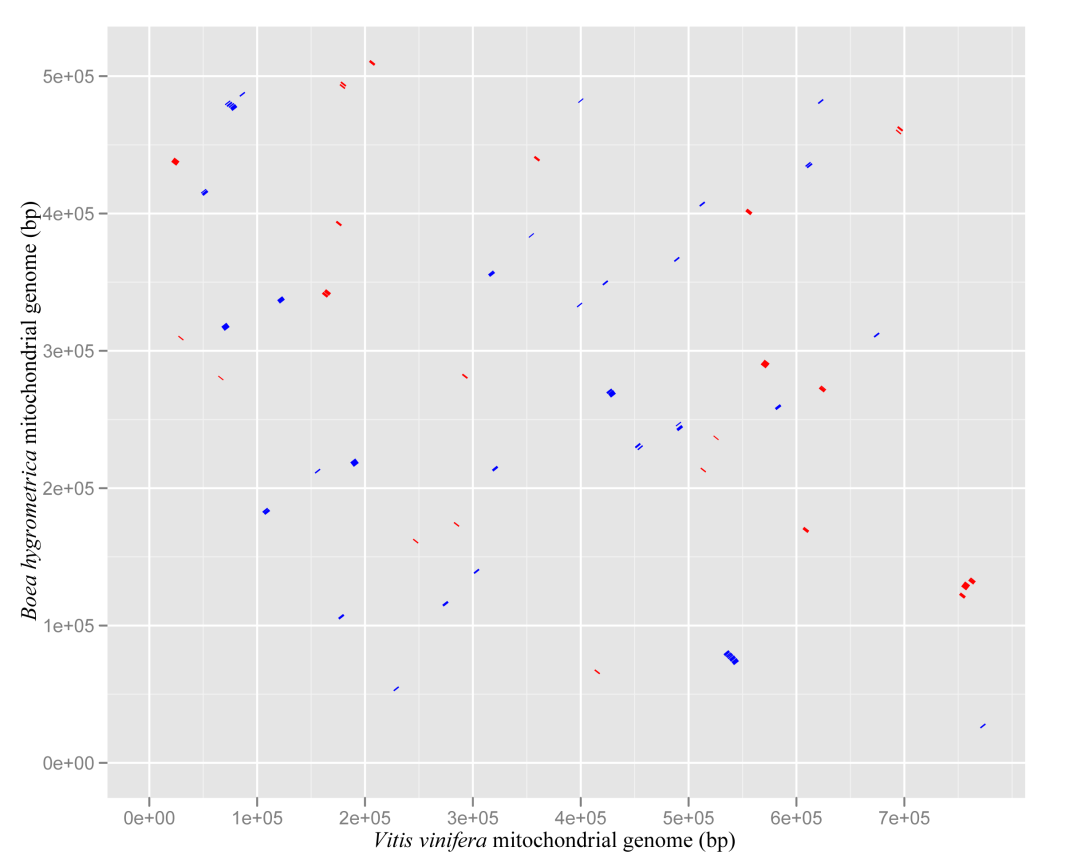


**Fig S3**
