# Supplementary material for: The Complete Chloroplast and Mitochondrial Genome Sequences of Boea hygrometrica: Insights into the Evolution of Plant Organellar Genomes
Source: PLoS One. 2012 Jan 23;7(1):e30531. doi: 10.1371/journal.pone.0030531 (PMC3264610; doi:10.1371/journal.pone.0030531)
Supplement: Figure S4 — The GC distribution between new and old cp-derived sequences in mitochondrial genome of Boea hygrometrica. Hits stand for the results of homologous alignments with other known mitochondrial genomes. (DOC) [file pone.0030531.s004.doc]

**S4.** The GC distribution between new and old cp-derived sequences in mitochondrial genome of *Boea hygrometrica*. Hits stand for the results of homologous alignments with other known mitochondrial genomes.

**
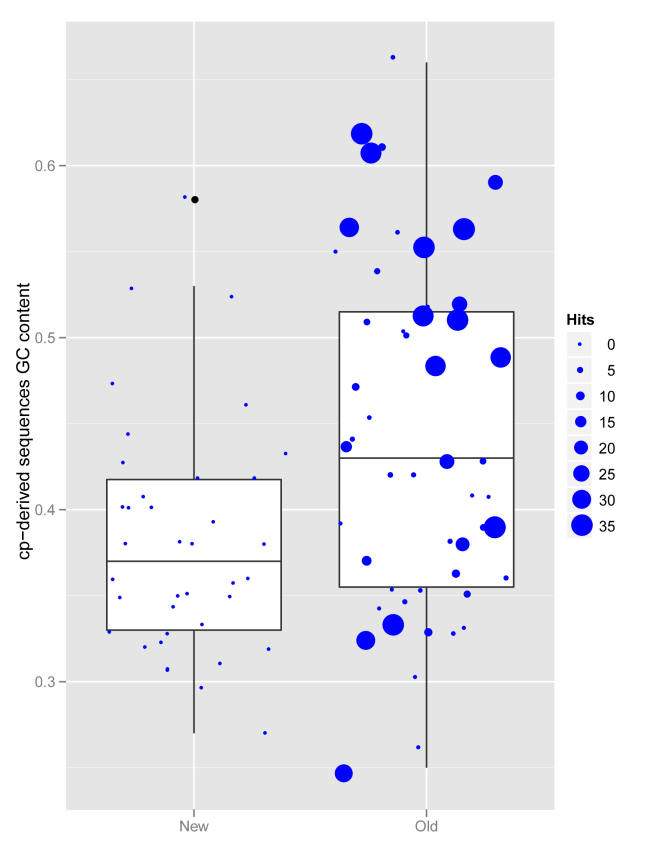
**

**Fig S4**
