# Supplementary material for: The Complete Chloroplast and Mitochondrial Genome Sequences of Boea hygrometrica: Insights into the Evolution of Plant Organellar Genomes
Source: PLoS One. 2012 Jan 23;7(1):e30531. doi: 10.1371/journal.pone.0030531 (PMC3264610; doi:10.1371/journal.pone.0030531)
Supplement: Table S1 — Table Codon usage table of the chloroplast genes. (DOC) [file pone.0030531.s005.doc]

**Table S1** Table Codon usage table of the chloroplast genes

| **Codon** | **Count** | **RSCU** | **Codon** | **Count** | **RSCU** | **Codon** | **Count** | **RSCU** | **Codon** | **Count** | **RSCU** |
| --- | --- | --- | --- | --- | --- | --- | --- | --- | --- | --- | --- |
| **UUU(F)** | 1016 | 1.28 | **UCU(S)** | 629 | 1.71 | **UAU(Y)** | 821 | 1.63 | **UGU(C)** | 221 | 1.41 |
| **UUC(F)** | 568 | 0.72 | **UCC(S)** | 359 | 0.97 | **UAC(Y)** | 187 | 0.37 | **UGC(C)** | 92 | 0.59 |
| **UUA(L)** | 895 | 1.83 | **UCA(S)** | 449 | 1.22 | **UAA(*)** | 45 | 1.21 | **UGA(*)** | 30 | 0.8 |
| **UUG(L)** | 585 | 1.2 | **UCG(S)** | 215 | 0.58 | **UAG(*)** | 37 | 0.99 | **UGG(W)** | 479 | 1 |
| **CUU(L)** | 644 | 1.32 | **CCU(P)** | 442 | 1.56 | **CAU(H)** | 478 | 1.5 | **CGU(R)** | 348 | 1.25 |
| **CUC(L)** | 200 | 0.41 | **CCC(P)** | 213 | 0.75 | **CAC(H)** | 159 | 0.5 | **CGC(R)** | 99 | 0.36 |
| **CUA(L)** | 415 | 0.85 | **CCA(P)** | 330 | 1.16 | **CAA(Q)** | 739 | 1.52 | **CGA(R)** | 384 | 1.38 |
| **CUG(L)** | 195 | 0.4 | **CCG(P)** | 150 | 0.53 | **CAG(Q)** | 234 | 0.48 | **CGG(R)** | 132 | 0.48 |
| **AUU(I)** | 1099 | 1.44 | **ACU(T)** | 575 | 1.64 | **AAU(N)** | 995 | 1.53 | **AGU(S)** | 440 | 1.19 |
| **AUC(I)** | 493 | 0.64 | **ACC(T)** | 254 | 0.73 | **AAC(N)** | 306 | 0.47 | **AGC(S)** | 120 | 0.33 |
| **AUA(I)** | 705 | 0.92 | **ACA(T)** | 414 | 1.18 | **AAA(K)** | 1076 | 1.45 | **AGA(R)** | 530 | 1.91 |
| **AUG(M)** | 660 | 1 | **ACG(T)** | 156 | 0.45 | **AAG(K)** | 410 | 0.55 | **AGG(R)** | 172 | 0.62 |
| **GUU(V)** | 546 | 1.49 | **GCU(A)** | 608 | 1.74 | **GAU(D)** | 901 | 1.61 | **GGU(G)** | 599 | 1.32 |
| **GUC(V)** | 172 | 0.47 | **GCC(A)** | 228 | 0.65 | **GAC(D)** | 215 | 0.39 | **GGC(G)** | 176 | 0.39 |
| **GUA(V)** | 544 | 1.48 | **GCA(A)** | 418 | 1.2 | **GAA(E)** | 1046 | 1.49 | **GGA(G)** | 717 | 1.58 |
| **GUG(V)** | 204 | 0.56 | **GCG(A)** | 142 | 0.41 | **GAG(E)** | 354 | 0.51 | **GGG(G)** | 328 | 0.72 |
