# Supplementary material for: The Complete Chloroplast and Mitochondrial Genome Sequences of Boea hygrometrica: Insights into the Evolution of Plant Organellar Genomes
Source: PLoS One. 2012 Jan 23;7(1):e30531. doi: 10.1371/journal.pone.0030531 (PMC3264610; doi:10.1371/journal.pone.0030531)
Supplement: Table S2 — cp and mt genomes of 15 plants comparison in this study. (DOC) [file pone.0030531.s006.doc]

**Table S2** cp and mt genomes of 15 plants comparison in this study

| **Claffication** | **Scientific Name** | **Common Name** | **Taxonomy IDs** | **Cp accession number** | **Mt accession number** | **Reference** |
| --- | --- | --- | --- | --- | --- | --- |
| **Algae** |  |  |  |  |  |  |
| Charophyta | *Chara vulgaris* | Green algae | 55564 | NC_008097 | NC_005255 |  |
| **Land plants** |  |  |  |  |  |  |
| Bryophyta | *Marchantia polymorpha* | Livewort | 3197 | NC_001319 | NC_001660 |  |
|  | *Megaceros aenigmaticus* | Hornworts | 13813 | NA | NC_012651 |  |
| **Seed plants** |  |  |  |  |  |  |
| Gymnosperm |  |  |  |  |  |  |
| Cycads | *Cycas taitungensis* | Emperor sago | 54799 | NC_009618 | NC_010303 |  |
| Angiosperm |  |  |  |  |  |  |
| Monocots | *Triticum aestivum* | Wheat | 4565 | NC_002762 | NC_007579 |  |
|  | *Oryza sativa* | Rice | 39947 | NC_001320 | NC_011033 |  |
|  | *Sorghum bicolor* | Sorghum | 4558 | NC_008602 | NC_008360 |  |
|  | *Tripsacum dactyloides* | Gama grass | 4563 | NA | NC_008362 |  |
|  | *Zea mays* | Maize | 381124 | NC_001666 | NC_007982 |  |
| Dicots | *Beta vulgaris* | Sugar beet | 3555 | NA | NC_002511 |  |
|  | *Brassica napus* | Oilseed rape | 3708 | NA | NC_008285 |  |
|  | *Arabidopsis thaliana* | Mouse-ear cress | 3702 | NC_000932 | NC_001284 |  |
|  | *Nicotiana tabacum* | Tobacco | 4097 | NC_001879 | NC_006581 |  |
|  | *Vitis vinifera* | Grape | 29760 | NC_007957 | NC_012119 |  |
|  | *Boea hygrometrica* | Xuan shuo ju tai (Pinyin, China) | 472368 | JN107811 | JN107812 |  |
|  | Olea europaea | olive | 4146 | NC_013707 | NA |  |

**Reference**

1. Turmel M, Otis C, Lemieux C (2003) The mitochondrial genome of Chara vulgaris: Insights into the mitochondrial DNA architecture of the last common ancestor of green algae and land plants. Plant Cell 15: 1888-1903.

2. Turmel M, Otis C, Lemieux C (2006) The chloroplast genome sequence of Chara vulgaris sheds new light into the closest green algal relatives of land plants. Molecular Biology and Evolution 23: 1324-1338.

3. Takemura M, Oda K, Yamato K, Ohta E, Nakamura Y, et al. (1992) Gene Clusters for Ribosomal-Proteins in the Mitochondrial Genome of a Liverwort, Marchantia-Polymorpha. Nucleic acids research 20: 3199-3205.

4. Shimada H, Sugiura M (1991) Fine structural features of the chloroplast genome: comparison of the sequenced chloroplast genomes. Nucleic Acids Res 19: 983-995.

5. Li LB, Wang B, Liu Y, Qiu YL (2009) The Complete Mitochondrial Genome Sequence of the Hornwort Megaceros aenigmaticus Shows a Mixed Mode of Conservative Yet Dynamic Evolution in Early Land Plant Mitochondrial Genomes. Journal of Molecular Evolution 68: 665-678.

6. Chaw SM, Shih ACC, Wang D, Wu YW, Liu SM, et al. (2008) The mitochondrial genome of the gymnosperm Cycas taitungensis contains a novel family of short interspersed elements, Bpu sequences, and abundant RNA editing sites. Molecular Biology and Evolution 25: 603-615.

7. Wu CS, Wang YN, Liu SM, Chaw SM (2007) Chloroplast genome (cpDNA) of Cycas taitungensis and 56 cp protein-coding genes of Gnetum parvifolium: insights into cpDNA evolution and phylogeny of extant seed plants. Molecular Biology and Evolution 24: 1366-1379.

8. Ogihara Y, Yamazaki Y, Murai K, Kanno A, Terachi T, et al. (2005) Structural dynamics of cereal mitochondrial genomes as revealed by complete nucleotide sequencing of the wheat mitochondrial genome. Nucleic acids research 33: 6235-6250.

9. Ogihara Y, Isono K, Kojima T, Endo A, Hanaoka M, et al. (2002) Structural features of a wheat plastome as revealed by complete sequencing of chloroplast DNA. Molecular Genetics and Genomics 266: 740-746.

10. Notsu Y, Masood S, Nishikawa T, Kubo N, Akiduki G, et al. (2002) The complete sequence of the rice (Oryza sativa L.) mitochondrial genome: frequent DNA sequence acquisition and loss during the evolution of flowering plants. Molecular Genetics and Genomics 268: 434-445.

11. Hiratsuka J, Shimada H, Whittier R, Ishibashi T, Sakamoto M, et al. (1989) The complete sequence of the rice (Oryza sativa) chloroplast genome: intermolecular recombination between distinct tRNA genes accounts for a major plastid DNA inversion during the evolution of the cereals. Mol Gen Genet 217: 185-194.

12. Saski C, Lee SB, Fjellheim S, Guda C, Jansen RK, et al. (2007) Complete chloroplast genome sequences of Hordeum vulgare, Sorghum bicolor and Agrostis stolonifera, and comparative analyses with other grass genomes. Theor Appl Genet 115: 571-590.

13. Clifton SW, Minx P, Fauron CMR, Gibson M, Allen JO, et al. (2004) Sequence and comparative analysis of the maize NB mitochondrial genome. Plant Physiology 136: 3486-3503.

14. Rodermel SR (1992) Nucleotide sequence of a maize chloroplast DNA fragment containing an inversion breakpoint, trnG (GCC), trnG (UCC), trnfM, and a trnG pseudogene. Nucleic Acids Res 20: 5844.

15. Kubo T, Nishizawa S, Sugawara A, Itchoda N, Estiati A, et al. (2000) The complete nucleotide sequence of the mitochondrial genome of sugar beet (Beta vulgaris L.) reveals a novel gene for tRNA(Cys)(GCA). Nucleic acids research 28: 2571-2576.

16. Handa H (2003) The complete nucleotide sequence and RNA editing content of the mitochondrial genome of rapeseed (Brassica napus L.): comparative analysis of the mitochondrial genomes of rapeseed and Arabidopsis thaliana. Nucleic acids research 31: 5907-5916.

17. Unseld M, Marienfeld JR, Brandt P, Brennicke A (1997) The mitochondrial genome of Arabidopsis thaliana contains 57 genes in 366,924 nucleotides. Nature Genetics 15: 57-61.

18. Sato S, Nakamura Y, Kaneko T, Asamizu E, Tabata S (1999) Complete structure of the chloroplast genome of Arabidopsis thaliana. DNA Res 6: 283-290.

19. Shinozaki K, Ohme M, Tanaka M, Wakasugi T, Hayashida N, et al. (1986) The complete nucleotide sequence of the tobacco chloroplast genome: its gene organization and expression. EMBO J 5: 2043 - 2049.

20. Sugiyama Y, Watase Y, Nagase M, Makita N, Yagura S, et al. (2005) The complete nucleotide sequence and multipartite organization of the tobacco mitochondrial genome: comparative analysis of mitochondrial genomes in higher plants. Molecular Genetics and Genomics 272: 603-615.

21. Goremykin VV, Salamini F, Velasco R, Viola R (2009) Mitochondrial DNA of Vitis vinifera and the Issue of Rampant Horizontal Gene Transfer. Molecular Biology and Evolution 26: 99-110.

22. Jansen RK, Kaittanis C, Saski C, Lee SB, Tomkins J, et al. (2006) Phylogenetic analyses of Vitis (Vitaceae) based on complete chloroplast genome sequences: effects of taxon sampling and phylogenetic methods on resolving relationships among rosids. Bmc Evolutionary Biology 6: 32.

23. Besnard G, Hernandez P, Khadari B, Dorado G, Savolainen V (2011) Genomic profiling of plastid DNA variation in the Mediterranean olive tree. Bmc Plant Biology 11.
