# Supplementary material for: The Complete Chloroplast and Mitochondrial Genome Sequences of Boea hygrometrica: Insights into the Evolution of Plant Organellar Genomes
Source: PLoS One. 2012 Jan 23;7(1):e30531. doi: 10.1371/journal.pone.0030531 (PMC3264610; doi:10.1371/journal.pone.0030531)
Supplement: Table S3 — Codon usage table of the mitochondrial genes. (DOC) [file pone.0030531.s007.doc]

**Table S3** Codon usage table of the mitochondrial genes

| **Codon** | **Count** | **RSCU** | **Codon** | **Count** | **RSCU** | **Codon** | **Count** | **RSCU** | **Codon** | **Count** | **RSCU** |
| --- | --- | --- | --- | --- | --- | --- | --- | --- | --- | --- | --- |
| **UUU(F)** | 423 | 1.17 | **UCU(S)** | 216 | 1.32 | **UAU(Y)** | 286 | 1.53 | **UGU(C)** | 95 | 1.13 |
| **UUC(F)** | 299 | 0.83 | **UCC(S)** | 158 | 0.97 | **UAC(Y)** | 88 | 0.47 | **UGC(C)** | 73 | 0.87 |
| **UUA(L)** | 302 | 1.55 | **UCA(S)** | 195 | 1.2 | **UAA(*)** | 20 | 1.4 | **UGA(*)** | 16 | 1.12 |
| **UUG(L)** | 219 | 1.13 | **UCG(S)** | 133 | 0.82 | **UAG(*)** | 7 | 0.49 | **UGG(W)** | 166 | 1 |
| **CUU(L)** | 240 | 1.23 | **CCU(P)** | 206 | 1.47 | **CAU(H)** | 189 | 1.45 | **CGU(R)** | 154 | 1.31 |
| **CUC(L)** | 124 | 0.64 | **CCC(P)** | 109 | 0.78 | **CAC(H)** | 71 | 0.55 | **CGC(R)** | 67 | 0.57 |
| **CUA(L)** | 188 | 0.97 | **CCA(P)** | 162 | 1.16 | **CAA(Q)** | 234 | 1.5 | **CGA(R)** | 148 | 1.26 |
| **CUG(L)** | 94 | 0.48 | **CCG(P)** | 82 | 0.59 | **CAG(Q)** | 78 | 0.5 | **CGG(R)** | 80 | 0.68 |
| **AUU(I)** | 386 | 1.33 | **ACU(T)** | 194 | 1.38 | **AAU(N)** | 274 | 1.38 | **AGU(S)** | 171 | 1.05 |
| **AUC(I)** | 245 | 0.85 | **ACC(T)** | 134 | 0.95 | **AAC(N)** | 123 | 0.62 | **AGC(S)** | 106 | 0.65 |
| **AUA(I)** | 237 | 0.82 | **ACA(T)** | 159 | 1.13 | **AAA(K)** | 304 | 1.23 | **AGA(R)** | 173 | 1.47 |
| **AUG(M)** | 292 | 1 | **ACG(T)** | 77 | 0.55 | **AAG(K)** | 189 | 0.77 | **AGG(R)** | 84 | 0.71 |
| **GUU(V)** | 201 | 1.23 | **GCU(A)** | 254 | 1.51 | **GAU(D)** | 252 | 1.4 | **GGU(G)** | 228 | 1.29 |
| **GUC(V)** | 121 | 0.74 | **GCC(A)** | 161 | 0.95 | **GAC(D)** | 108 | 0.6 | **GGC(G)** | 95 | 0.54 |
| **GUA(V)** | 195 | 1.19 | **GCA(A)** | 176 | 1.04 | **GAA(E)** | 311 | 1.42 | **GGA(G)** | 254 | 1.44 |
| **GUG(V)** | 136 | 0.83 | **GCG(A)** | 84 | 0.5 | **GAG(E)** | 128 | 0.58 | **GGG(G)** | 128 | 0.73 |
